# Supplementary material for: Alterations in gut microbiota and host transcriptome of patients with coronary artery disease
Source: BMC Microbiol. 2023 Nov 3;23:320. doi: 10.1186/s12866-023-03071-w (PMC10623719; doi:10.1186/s12866-023-03071-w)
Supplement: Supplementary file 2 — Additional file 2: Supplementary Figure S1. Differential of gut microbiota between CAD patients and controls at class (A), order (B), family (C), and species (D) level. The abundance of microbe was log-transformed. The significant level of each microbe was calculated by using Student’s t-test. (E) The top 10 differential genera between CAD and control group by means of Mean Decrease Gini. [file 12866_2023_3071_MOESM2_ESM.pdf]

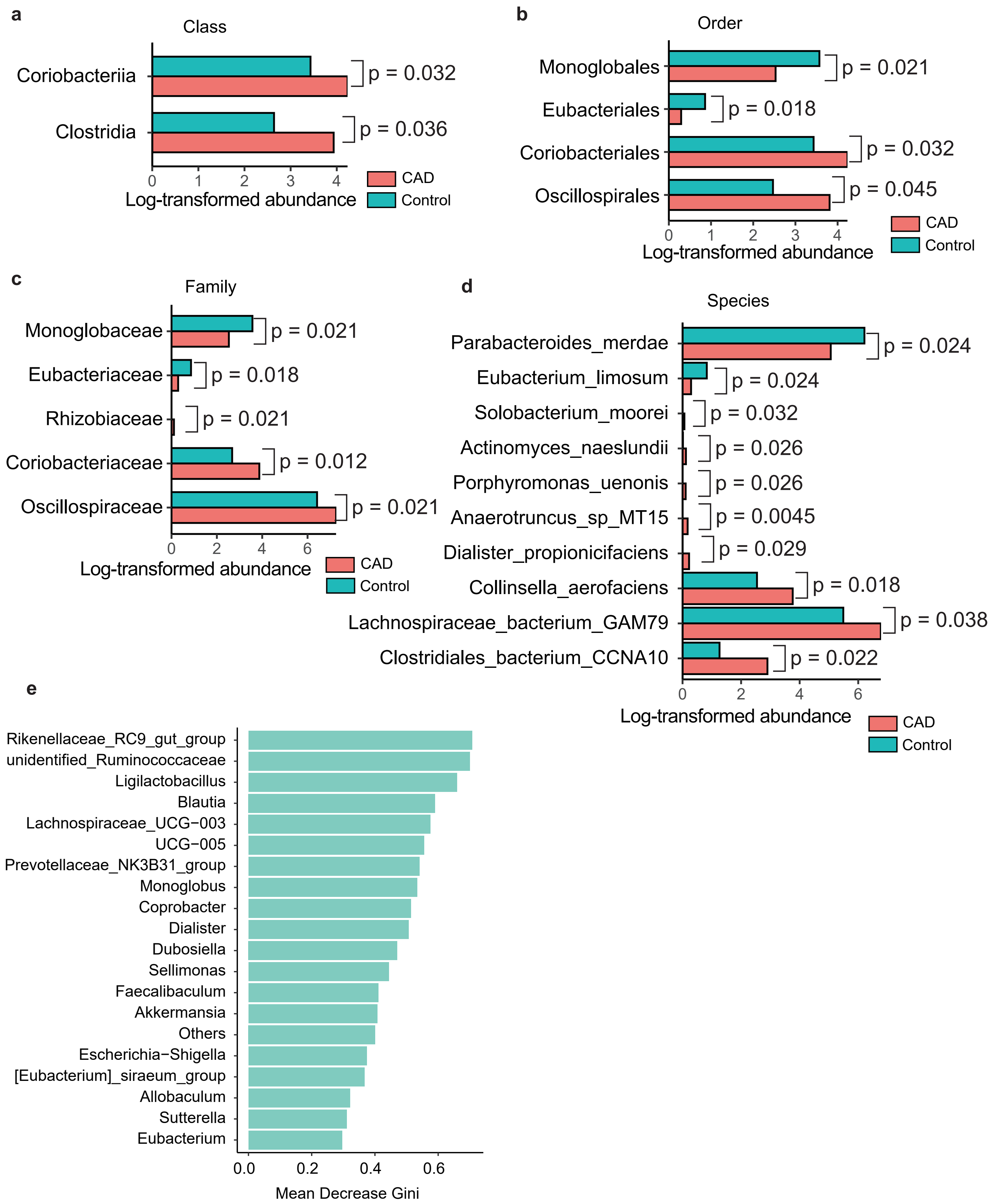

Supplementary Figure S1. Differential of gut microbiota between CAD patients and controls at class (A), order (B), family (C), and species (D) level. The abundance of microbe was log-transformed. The significant level of each microbe was calculated by using Student's t-test. (E) The top 10 differential genera between CAD and control group by means of Mean Decrease Gini.
