# Supplementary material for: Alterations in gut microbiota and host transcriptome of patients with coronary artery disease
Source: BMC Microbiol. 2023 Nov 3;23:320. doi: 10.1186/s12866-023-03071-w (PMC10623719; doi:10.1186/s12866-023-03071-w)
Supplement: Supplementary file 3 — Additional file 3: Supplementary Figure S2. The association of differential abundant bacteria with clinical indices accordining to different levels of order (A), family (B), and species (C). Significances tested by Pearson correlation analysis. * FDR < 0.05; ** FDR < 0.01; *** FDR < 0.001. Color bar indicates the level of correlation coefficients (corr). [file 12866_2023_3071_MOESM3_ESM.pdf]

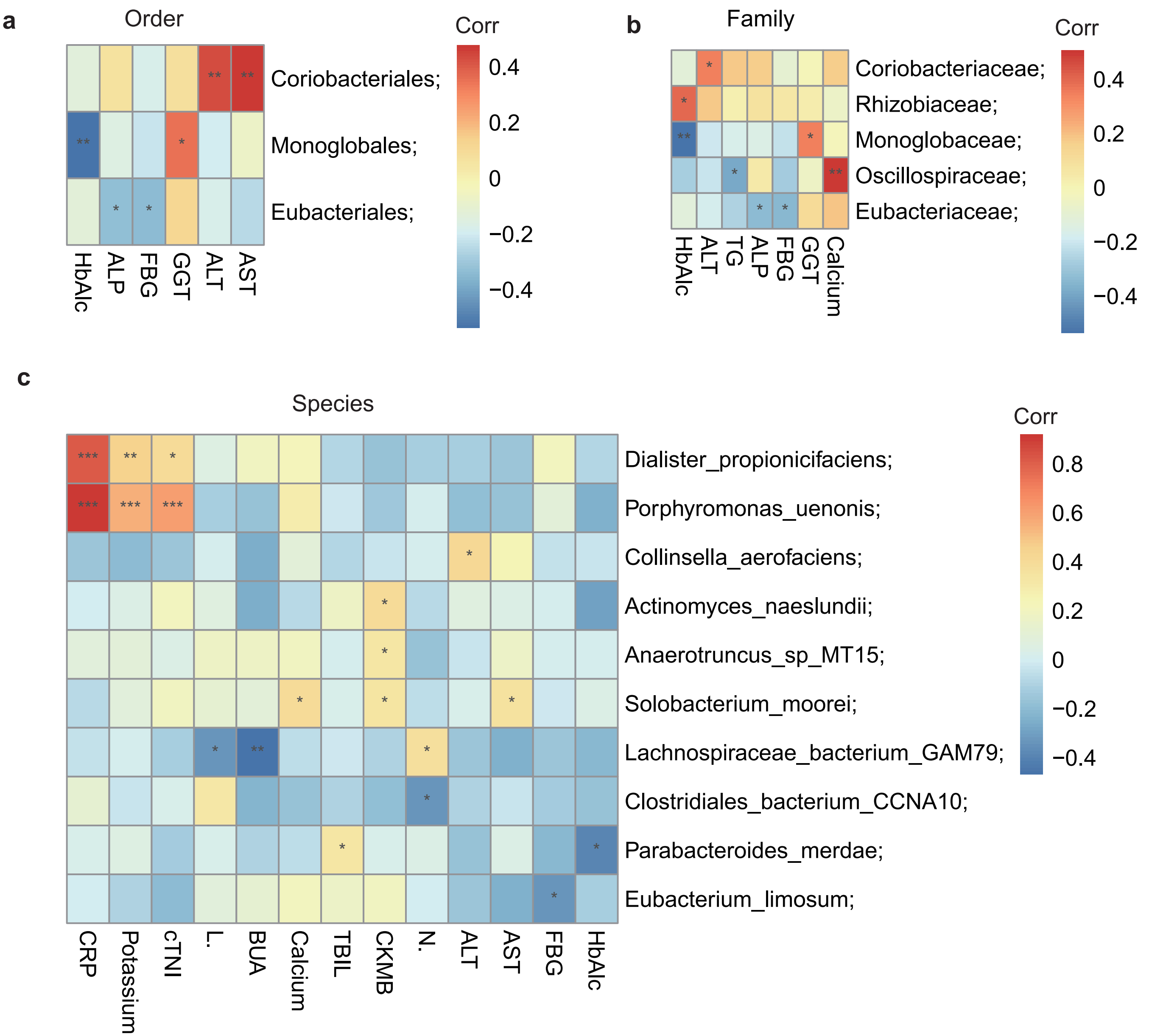

Supplementary Figure S2. The association of differential abundant bacteria with clinical indices accordining to different levels of order (A), family (B), and species (C). Significances tested by Pearson correlation analysis. * FDR < 0.05; ** FDR < 0.01; *** FDR < 0.001.
Color bar indicates the level of correlation coefficients (corr).
